# Innovative applications of 3D printing in personalized medicine and complex drug delivery systems

**DOI:** 10.1016/j.isci.2025.113505

**Published:** 2025-09-05

**Authors:** Devesh U. Kapoor, Anil Pareek, Priyanka Uniyal, Bhupendra G. Prajapati, Kasitpong Thanawuth, Pornsak Sriamornsak

**Affiliations:** 1 Dr. Dayaram Patel Pharmacy College, Bardoli, Gujarat 394601, India; 2 Department of Pharmaceutics, Lachoo Memorial College of Science and Technology (Autonomous), Jodhpur, Rajasthan 342001, India; 3 School of Health Sciences and Technology, UPES, Dehradun, Uttarakhand 248007, India; 4 Department of Pharmaceutics, Parul Institute of Pharmacy, Faculty of Pharmacy, Parul University, Vadodara, Gujarat 391760, India; 5 Centre for Research Impact & Outcome, Chitkara College of Pharmacy, Chitkara University, Rajpura, Punjab 140401, India; 6 College of Pharmacy, Rangsit University, Pathum Thani 12000, Thailand; 7 Department of Industrial Pharmacy, Faculty of Pharmacy, Silpakorn University, Nakhon Pathom 73000, Thailand; 8 Academy of Science, The Royal Society of Thailand, Bangkok 10300, Thailand

**Keywords:** Health sciences, Biological sciences, Bioengineering, Biotechnology

## Abstract

Three-dimensional (3D) printing, or additive manufacturing, is transforming pharmaceutical and biomedical fields by enabling personalized medicine. This review highlights advances in 3D printing for customized drug delivery systems, including patient-specific dosage forms, multidrug polypills, and implantable devices that improve adherence and therapeutic outcomes—especially for pediatric and geriatric populations. The intersection of 3D printing with regenerative medicine is also explored, focusing on bioprinting technologies, stem cell-laden scaffolds, and smart biomaterials such as hydrogels and bioinks for localized therapy and tissue repair. These strategies reflect an expanded vision of personalized medicine, merging individualized pharmacotherapy with tissue engineering. Additionally, the review discusses the integration of artificial intelligence, nano-enabled platforms, and decentralized pharmaceutical manufacturing to accelerate development and access. Key regulatory and technical challenges are outlined, along with future directions to promote the clinical translation and scalability of 3D-printed pharmaceutical and regenerative systems.

## Graphical abstract

**Figure:**
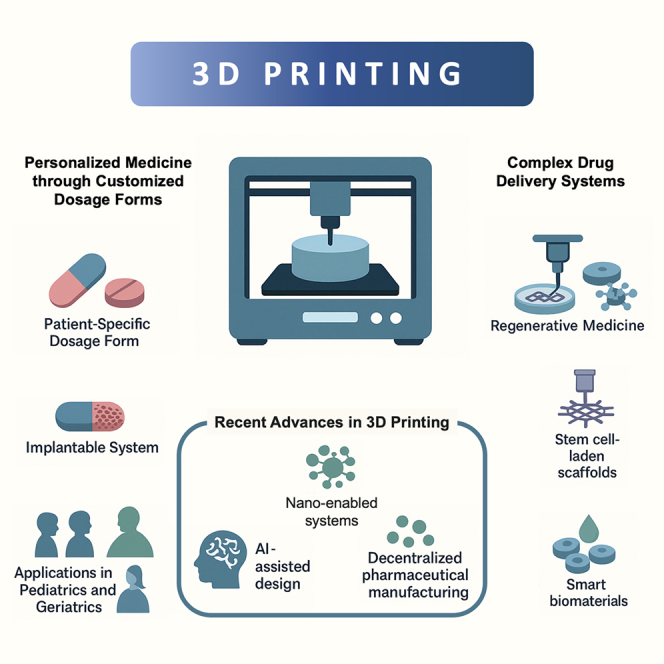

## Introduction

Three-dimensional (3D) printing technology allows the construction of intricate structures by the continuous deposition of material layers based on a digital model, with each layer helping to produce the final product until it is completely formed.^1^^,^^2^^,^^3^ In the pharmaceutical sector, 3D printing has attracted considerable attention, with specialists forecasting its potential to markedly enhance dosage form production.^4^^,^^5^ The enhancement of 3D printing techniques enables the production of high-quality dosage forms with diverse drug release patterns. Various 3D printing methodologies are used in the pharmaceutical industry, such as fused deposition modeling (FDM), selective laser sintering (SLS), stereolithography (SLA), inkjet-based 3D printing, and pressure-assisted microsyringe (PAM) printing. The choice of a suitable 3D printing method for manufacturing certain dosage forms is determined by the nature of active pharmaceutical ingredients (APIs) and polymers, together with the intended tablet shape and release properties. The use of 3D printing in medicine has grown since the 1980s. Web of Science reports a substantial rise in publications including the phrases “3D printing” and “drug” in the “pharmacy/pharmacology” category, rising from 14 articles in 2009 to 134 in 2019. This developing material of research indicates increased interest in 3D printing for therapeutic purposes, particularly in the United Kingdom and the United States. Among its advantages, 3D printing technology remains a new tool for the modification and personalization of pharmaceutical products. Unlike traditional tablet production processes, 3D printers give flexibility that enables pharmaceutical businesses to send digital blueprints of their compositions to local pharmacies and healthcare institutions.^6^^,^^7^ This allows the "on-demand" creation of tailored pharmaceuticals, overcoming the constraints of the old “one-size-fits-all” method.^8^ Additionally, 3D printing’s capacity to customize pharmaceuticals might turn ordinary pharmacies into digital pharmaceutical manufacturing units.^9^

Utilizing computer-aided design (CAD) data, 3D printing permits pharmacists to generate personalized formulas immediately, altering designs as required to fit individual patient needs.^10^ This customization enables the manufacture of pharmaceutical solutions matched to the age, body weight, organ condition, and degree of sickness.^11^ Furthermore, the realm of personalized medicine (PM) encompasses not only customized medication doses but also patient-specific tissue regeneration and implanted therapeutic devices. This advancement has established 3D bioprinting, where live cells and biomaterials are fabricated into functional structures as an essential instrument in developing regenerative remedies that enhance personalized medication treatment.^12^^,^^13^ In addition, 3D printing allows the fabrication of single-dose forms combining various APIs, which is particularly helpful for patients with different illnesses, since it decreases the need for many different drugs.^14^^,^^15^ Furthermore, for individuals who have difficulties swallowing, 3D-printed tablets may be tailored to fast dissolve, coinciding with particular patient pref.^6^ The flexibility of 3D printing also enables the development of solid dosage forms^16^ with complex designs suited for managing drug release rates.^8^^,^^17^ Considering these benefits, 3D printing presents problems compared to conventional pharmaceutical manufacturing procedures, especially for large-scale production. In this regard, high-speed tablet printers are capable of manufacturing up to 240,000 tablets per hour.^18^ As a result, the first FDA-approved 3D-printed medicine, Spritam (levetiracetam), could only be manufactured in numbers of tens of thousands of units per hour, mostly owing to the layer-by-layer manufacturing method, which is time-intensive (Cairns, 2018). This gap in production speed restricts the utility of 3D printing for mass manufacture; nevertheless, 3D printing remains acceptable for small-scale production in pharmaceutical settings where speed limitations are less crucial. For commercialized pharmaceutical production employing 3D printing, fulfilling the U.S. Food and Drug Administration recommendations on development, production considerations, and device validation is necessary.^19^ However, assuring uniform quality among decentralized 3D printing establishments is challenging, since the FDA cannot directly supervise every individual printing operation. The differences in printer layout, material suppliers, software, and production procedures may lead to discrepancies and product faults. Therefore, if a 3D-printed dosage form is manufactured with faults, there is potential for patient damage.^20^ Furthermore, the mass manufacturing of 3D printing medications by unauthorized individuals or groups possessing access to drug development may be a violation of patent rights. Mass production utilizing traditional procedures requires less energy and less time to manufacture a broad range of dosage forms, but small batches made using 3D printing are more affordable than conventional production techniques.^21^ Although this technology is expensive, its application will reduce expenses and make pharmaceutical drugs more affordable compared to their equivalent dosage forms created using traditional pharmaceutical methods. Despite the tremendous potential of 3D printing in personalized healthcare, its integration into routine clinical practice remains limited. Key advantages of this technology include the ability to tailor medication doses to individual patient attributes, combine multiple pharmaceuticals into a single dosage form, and fabricate on-demand formulations with complex release profiles. However, several challenges persist. These include issues of scalability for mass production, regulatory standardization, limited availability of pharmaceutically suitable printing materials, and variability among decentralized manufacturing units. Moreover, there remains an insufficient understanding of the long-term stability, bioavailability, and patient outcomes associated with 3D-printed pharmaceutical products.

Current research largely focuses on individual aspects such as material science or regulatory pathways, leaving a gap in integrative studies that connect technological innovations to real-world clinical deployment. This review aims to address this gap by providing a unified perspective on how 3D printing technologies are driving advancements in PM across both pharmacological and regenerative domains. It provides a comprehensive and cohesive overview, linking personalized drug delivery systems with cutting-edge developments in 3D bioprinting and regenerative medicine. The review highlights how 3D printing enables customized dosage forms and opens new avenues in tissue engineering, stem-cell therapies, and bioink development—key components of personalized healthcare.

Unlike existing reviews that primarily focus on isolated topics such as drug release kinetics, regulatory pathways, or material science, this work uniquely integrates applications across pediatrics, geriatrics, and polypill formulations. It further extends into advanced areas including bioprinting, stem-cell integration, and smart polymer-based systems. The article also presents detailed case studies, examines the clinical implications of patient-specific dosage forms, and showcases innovations such as 3D-printed microneedles, implants, and scaffolds.

Additionally, emerging bioinks and hydrogels in tissue regeneration are explored alongside discussions on regulatory challenges and the transformative role of AI, nanoprinting, and decentralized drug manufacturing in shaping the future landscape. This multifaceted approach, spanning material innovation to patient-centered applications, provides a broader and more clinically relevant perspective than the narrower focus of existing literature.

## Personalized medicine through 3D printing

### Customized dosage forms

PM involves developing treatments to meet the individual needs, characteristics, and preferences of participants.^22^ Personalizing pharmaceuticals according to the unique characteristics of individuals is strongly advised in the current situation (e.g., physical, physiological, and clinical). Conversely, personalizing medicines for a diverse population with multiple variances could bring significant challenges. Moreover, individuals with comorbidities have problems adapting to devices and drugs for therapy to varying degrees.^23^ The customization of medications has acquired considerable importance by resolving many limitations. Therefore, PM functions as a technique that prevents, diagnoses, and treats human medical issues based only on the genes of an individual.^24^ Table 1 summarizes the different 3D printing techniques used in pharmaceutical applications.

**Table 1:** Summary of current 3D printing techniques used in pharmaceutical applications

3D printing method	Materials used	Design strategies	Drug delivery mechanism	Advantages	Limitations	Reference
Fused deposition modeling (FDM)	Thermoplastic polymers (e.g., PVA, PLA)	Layer-by-layer deposition	Immediate and sustained release	Cost-effective, scalable	Limited to thermally stable drugs	Pervaiz et al.^25^
Inkjet printing	Drug solutions/suspensions, polymers	Drop-on-demand	Localized drug delivery	High resolution, low waste	Limited to low-viscosity formulations	Majrashi et al.^26^
Stereolithography (SLA)	Photosensitive resins	UV-curing layer-by-layer	Controlled release	High precision and complexity	Limited biocompatible resins	Zhang et al.^27^
Selective laser sintering (SLS)	Powdered polymers	Laser sintering of layers	Modified drug release	No need for binders	High temperature may degrade drugs	Han et al.^28^

**Figure 1:**
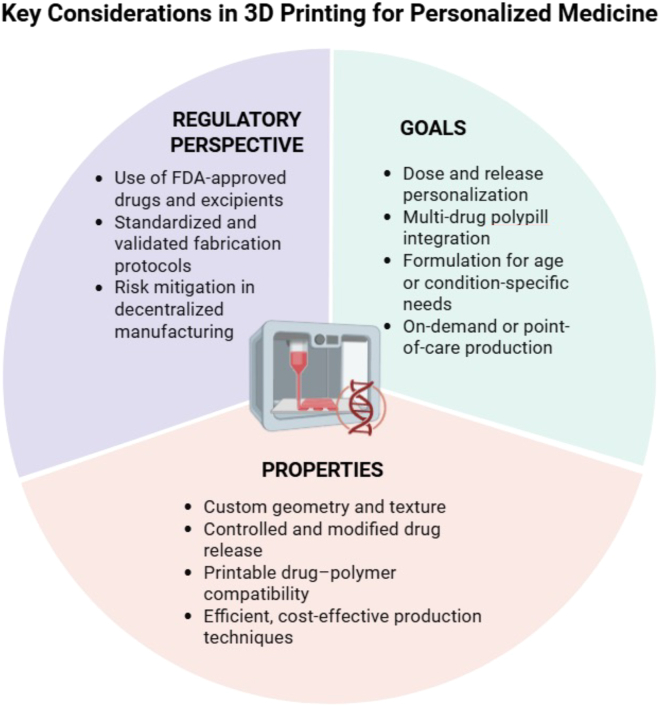
Key considerations in 3D printing for personalized medicine

In the pharmaceutical industry, this concept may be used by a certain group of persons who do not react to medicinal treatments as the broader market does.^29^ Figure 1 defines the distinct aims, desirable attributes, and regulatory considerations of personalized medications. Since customization relies on the DNA pattern of an individual, new technologies such as 3D printing may transform conventional production methods for future dosage forms. The finding that people react substantially to the same disease or ailment is due to chromosomal-assisted advancements in genomics and therapeutic investigations.^30^^,^^31^ 3D printing may change the market by boosting patient engagement, which might be done through immediate manufacture to give the ideal therapeutic care. In these cases, the created prototype should be flexible, adaptable, and customizable to the expectations and requirements of a patient while conforming to regulatory authorities.^4^^,^^32^

The "polypill," a formulation that combines several APIs in a single dosage form, is a recognized instance of 3D printing in PM. Every ingredient of the formulation has a unique therapeutic function. The polypill approach seeks to utilize the effectiveness of combination therapy to improve therapeutic results while minimizing production, delivery, and storage expenses.^33^ Despite this, traditional manufacturing procedures for these specific substances are problematic, especially since the standard "one-size-fits-all" strategy restricts customized therapeutic applications.^34^ Considering that different patient populations, such as children, adults, and elderly patients, have different needs for dosage and bioavailability. It is frequently required to use particular formulations with customized compositions and dosages.^35^ 3D printing, a manufacturing method, provides benefits such as minimized manufacturing processes, cost efficiency, and design versatility, which are especially advantageous for the on-demand development of personalized medications containing multiple APIs.^36^^,^^37^^,^^38^^,^^39^ Khaled et al. constructed a polypill utilizing 3D printing that included five cardiovascular drugs with varied geometries and release patterns, proving the ability to produce complicated, multi-drug systems in a single tablet.^37^ Although APIs in these dosage forms display different release kinetics^40^ and absorption locations, customizable release patterns in these multi-drug combinations are particularly helpful in satisfying clinical treatment needs. A study by Sadia et al. manufactured multi-layered tablets utilizing SLS 3D printing containing various APIs with compartmentalized release profiles for hypertension control, illustrating how such systems may minimize pill burden and increase therapeutic compliance.^41^ These issues must be properly studied while building compound compositions utilizing 3D printing technology. In the case of the management of type 2 diabetes, when metformin is largely absorbed in the intestinal tract and glimepiride in the digestive tract, Gioumouxouzis et al. applied hot-melt extrusion FDM 3D printing to construct an anti-diabetic dual-layer manufacturing. This dosage form has unique release characteristics for glimepiride and metformin, enabling concurrent, weekly administration.^42^ 3D printing can transform combination treatments by allowing the development of single tablets^43^ carrying several APIs, each having a distinct release profile, thereby improving medication regimes. However, thorough clinical control trials are important to ascertain the safety of these prescription drugs by examining possible drug-drug and drug-polymer interactions.^44^^,^^45^^,^^46^

### Applications in pediatrics and geriatrics

Children deal with special challenges in medicine administration owing to their distinct preferences about dose form, taste, texture, or scent, frequently leading to failure if these elements are unpleasant. While oral administration is usually easy, it may become complex for children, since they may reject medicine based on qualities such as shape, color, or flavor. Here, 3D printing provides a solution by responding to individual desires.^47^ A significant part of pediatric therapy is giving dosages adjusted to body weight, which 3D printing may also handle.^47^ For young children who may have swallowing difficulties, 3D printing compositions such as fast-dissolving tablets, orally disintegrating films (ODFs), and mini-tablets are acceptable. Recent research has shown the effective use of semi-solid extrusion 3D printing to create child-friendly orodispersible formulations, such as propranolol hydrochloride gummies, adapted to pediatric dosage demands.^48^ Additionally, amlodipine chewable tablets were produced for on-demand compounding in hospital pharmacies, offering a realistic option for age-appropriate, taste-masked pediatric medicines.^49^ Studies demonstrate that children favor mini-tablets of roughly 4 mm diameter than other formulations^50^ and that ODFs are more attractive than oral powders in unit dosage sachets in pediatrics.^51^ Personalizing dose forms with desired tastes and colors might further increase adherence. In one research, Goyanes et al. employed 3D printing to build chewable isoleucine pills in flavors (lemon, raspberry, coconut, and so forth) and colors (light green, yellow, orange, and so forth) matched children’s interests for maple syrup urine ailments, which were highly accepted.^52^ Another research successfully generated child-friendly chewable chocolate-based dose forms in varied sizes.^53^

In the majority of older individuals, swallowing pills becomes a substantial issue that might impact prescription adherence since swallowing difficulties increase with age. This may be rectified by employing quick disintegration tablets and ODF compositions, which can be manufactured by 3D printing. The senior population suffers from many diseases and needs multiple prescriptions and extended therapy, which brings in the concerns of polypharmacy.^54^ This practice may be handled using poly-pills created based on the patient’s demand via 3D printing. Many of them also experience cognitive impairment (dementia), which might compromise drug adherence. This may be addressed by 3D-printed dosage forms with imprinting patterns on them, which can show the date, time, or/and weekday for administration, accessible to each patient.^55^ In addition to tactile imprinting, recent research has investigated binder jet 3D printing for putting QR codes directly into tablets, enabling visible and scannable pharmaceutical advice. This method is particularly promising for older individuals with cognitive impairment, offering customized, legible signals that improve adherence.^56^

### Timeline of technological advances in 3D-printed drug delivery

Personalized delivery techniques involve controlled release,^57^ targeted administration, and flexible doses. These systems allow drugs to be delivered in response to precise triggers or physiological signals, assuring maximum therapeutic effectiveness while reducing possible negative effects.^58^ Research findings have revealed that 3D-printed dose forms have been widely approved by patients. Most people expressed favorable perspectives toward pharmaceuticals manufactured by 3D printers.^52^^,^^55^^,^^59^ In the early eighties, Charles Hull developed 3D printing technology, originally employed in engineering and different non-clinical production domains, including the automobile, aerospace, and household goods sectors. Since 2012, its applicability has increased dramatically. Significant advances in 3D printing techniques and an abundance of flexible, biocompatible components have permitted its widespread usage in the pharmaceutical industry.^60^^,^^61^ The creation of 3D printing breakthroughs relevant to pharmaceuticals started in the early nineties at the Massachusetts Institute of Technology (MIT, Cambridge, MA), where Sachs et al. developed and registered a rapid-prototyping approach referred to as “three-dimensional printing approaches.”^62^ This technology confirmed 3D printing as a feasible strategy in pharmaceutics, allowing the creation of a broad variety of pharmaceutical products, among them comprising poorly water-soluble medicines and proteins.^63^

The latest FDA authorization of the 3D-printed pharmaceutical substance Spritam generates substantial attention to 3D printing technologies, which is projected to alter pharmaceutical research, especially in PM.^64^ In general, 3D printing includes the accurate, layer-by-layer application of components using computer-generated patterns, allowing the production of sophisticated three-dimensional components. Primarily created in the 1980s, 3D printing used to be implemented for prototyping in sectors such as the automobile and aerospace. Whereas the FDA approval of Spritam in 2015 represented a key milestone for the use of 3D printing in therapeutics. Among the many 3D printing approaches, FDM has achieved significance.^58^^,^^64^ Conventional tablet production in the drug industry usually employs a “one-size-fits-all” strategy according to phase 3 clinical studies. Yet, this strategy may lead to inadequate doses, possibly leading to toxicities, adverse events, or diminished therapeutic effectiveness. Whereas 3D printing provides a solution via the targeted material application and precise control over factors including API segregation in combination treatments.^65^^,^^66^ The benefits of 3D printing over traditional approaches include the opportunity for personalization, enhanced structural complexity, and on-demand manufacture. This technique permits dosage personalization based on individual factors, such as body mass index, metabolism, and genetic variation, which may increase adherence to treatment. Advanced compositions, including multi-drug dosages, may also be generated, boosting therapeutic effectiveness. Latest studies have revealed the possibility of 3D printing in innovative delivery strategies. In particular, microneedle patches constructed via SLA 3D printing for the administration of insulin suggested promising preclinical outcomes in diabetic rodents. It was obtaining rapid plasma glucose decline within 1 h and maintaining insulin impacts for a maximum of 4 h, surpassing traditional subcutaneous delivery of insulin.^67^ A further study indicated that SLA-printed microneedles successfully allowed the transdermal distribution of compounds with varying molecular weights (FITC-Dextran and calcein) via full-thickness human skin *in vitro*, greatly improving absorption over untreated skin.^68^

## Complex drug delivery systems

### Innovative drug delivery mechanisms

3D printing technology has considerable potential for generating individualized pharmaceuticals and drug delivery systems, mainly due to personalized structural designs. Several studies have utilized 3D printing to change shell properties such as units, thickness, and gaps, to construct personalized printlets permitting adaptive drug release patterns. Arafat et al.^66^ designed a pharmaceutical formulation containing unique built-in gaps with repeated units, bridges, and gaps to allow fast release. Their optimal design, consisting of 9 units coupled by 3 bridges to generate 8 gaps, increased tablet release efficiency. Trials indicated that the resulting product provided a quick drug release rate of 86.7% within 30 min, fulfilling USP criteria for rapid release. Following this phase, the polymer matrix degraded at 8 μm/min, whereas hydroxypropyl cellulose enlarged, supporting release.^69^^,^^70^ Tan et al.^71^ supported generating tablets by ideally designed 3D-printed molds in different shapes and sizes, enabling the therapeutic agent to possess a regulated one-dimensional release profile, as dictated by the structure of the mold. These printlets include three different parts: a drug-free matrix, a drug-containing matrix, and a non-permeable, biodegradable covering layer. Upon contact with a dissolving liquid, the surface-dissolvable matrix facilitates layer-by-layer breakdown using the open portion of the tablet, which causes a release profile controlled by the developing matrix structure. By changing this surface-degradable matrix, different release patterns such as fast, delayed, ongoing, and moderate release can be produced.

3D printing allows the development of complex patterns, making it viable to manufacture tailored medical devices and oral dose forms.^52^^,^^72^ Although its development speed is slower than conventional drug production methods, 3D printing provides benefits including personalization and cost-efficiency for small-batch manufacturing.^73^ High-precision 3D printing techniques, including digital light processing (DLP) and stereolithography (SLA), enable the manufacture of small-scale drug delivery systems, known as microneedles (MNs).^74^ In recent years, Khaled et al. established the capacity of 3D printing to develop large-dose paracetamol tablets, which is problematic with traditional production, caused by constraints in material mixing and encapsulation.^75^ Microneedle arrays involve little needle-like structures that pass through the epidermis, increasing medication delivery while protecting skin integrity and minimizing infection risk compared with hypodermic injections. Modern additive manufacturing techniques such as SLA enhance the patch manufacturing operation by simplifying the fabrication of patches containing various medications with complicated designs that govern drug release patterns.^76^ High-resolution additive manufacturing techniques have been utilized to fabricate microneedles with precision and efficiency, involving a diverse array of materials.^77^ Inkjet printing, another additive manufacturing technique, is employed to achieve an identical drug coating on microneedles with precise and consistent dosing.^78^

The advancement of nanotechnologies has also been aided by 3D printing technology. Although aggregates may affect structural integrity, the arrangement of nanoparticles within 3D-printed materials is crucial. Particle distribution in liquid suspensions can be enhanced by pretreatment procedures such as ball milling, the addition of surfactant, and ultrasonic utilization.^79^ Polymeric polycaprolactone (PCL) nanocapsules contain redispersible 3D-printed solid dosage forms composed of polyphenols (such as curcumin and resveratrol). PAM incorporated these polyphenols into a carboxymethyl cellulose 3D-printed hydrogel, resulting in a significant release across 8 h. However, specific substances encapsulated in nanocapsules did not release, indicating a problem that needs to be addressed.^80^^,^^81^ A 3D-printed dissolved polymer scaffold of polylactic acid (PLA) and polyvinyl alcohol (PVA) was used to produce solid lipid compositions. The scaffold had different parts for a second-step solid lipid composition. Depending on the lipid formulation, emulsions of Gelucire 44/14, Kolliphor P188, Gelucire 48/16, and packed with model drugs, including fenofibrate, clofazimine, lumefantrine, and halofantrine, produced distinct release properties.^82^ Additionally, tablets containing solid self-nanoemulsifying drug delivery systems (SNEDDS) were effectively printed. Capryol 90, dapagliflozin, PEG 6000^83^ and 400, Poloxamer 188, and Cremophor EL were combined to produce a semisolid paste. The solid matrix contained surfactants, and the liquid phase of the lipid system consisted of oils and cosurfactants. Following the melting of the drugs and excipients, the combined mixture was transported to a PAM capsule for 3D printing. For dapagliflozin, this SNEDDS 3D-printed tablet showed an immediate release profile (>75% in 20 min).^84^ Additionally, PAM was used to 3D-print SNEDDS suppositories consisting of lidocaine to relieve hemorrhoids.^85^

### Implants and scaffolds

Drug-loaded implants may transport the active pharmaceuticals to the site of action effectively.^86^^,^^87^ Implants using pre-designed patterns have the benefit of delivering medications in elevated concentrations over longer durations.^88^ A unique void bullet-shaped implant with a porous surface facilitated the localized administration of chemotherapy drugs. This implant, produced using FDM 3D printing with PLA, was infused with the anticancer agent cyclophosphamide with an immersion method, resulting in accelerated drug release, which was decreased by the application of a PLA coating.^89^ In a further study, a four-layered central cylindrical implant composed of poly-D,L-lactic acid (PDLLA) was fabricated by inkjet-based 3D printing. Every layer alternated between levofloxacin and tobramycin, initiating drug release from the peripheral layer and promoting within, therefore enhancing bone marrow cell formation and regulating inflammation in a rabbit model of chronic osteomyelitis.^90^ Hollow cylindrical implants were fabricated by means of an FDM 3D printer from poly-L-lactide (PLLA), PCL, Eudragit RS PO, and ethyl cellulose, with quinine release varying based on polymer hydrophilicity and drug load.^91^ Nitrofurantoin-loaded PLA and HPMC implants were developed to promote drug distribution, with HPMC creating a porous network that facilitated drug release.^92^

Several 3D-printed scaffolds have shown potential for bone tissue recovery.^93^^,^^94^ One research revealed alendronate-loaded macro-porous scaffolds composed of β-TCP with a PCL covering, providing a controlled drug release for a period of seven days compared to the burst release from uncoated scaffolds. This technique conserved the structural integrity of β-TCP, promoting bone formation and permitting targeted drug administration during early wound healing.^95^ An additional study generated PCL-based scaffolds filled with cefazolin for targeted prophylaxis in surgical locations. With FDM coupled with salt leaching, these scaffolds developed macro/microstructural characteristics that facilitated drug loading. A gelatin methacrylate coating significantly decreased burst release, permitting regulated drug release for three days.^96^ Furthermore, a chitosan-pectin hydrogel scaffold comprising lidocaine hydrochloride was produced utilizing extrusion-based 3D printing and lyophilized for wound therapy. This scaffold demonstrated outstanding flexibility, adhesion strength to skin, and simple removal without tissue injury. It accomplished an initial burst release that follows continuous drug release for over 6 h, making it appropriate for wound therapy.^97^

### Bioprinting for regenerative medicine

#### 3D bioprinting

3D bioprinting has revolutionized regenerative medicine by enabling the creation of tissue-engineered constructs that integrate controlled drug delivery systems.^98^ These constructs address critical healthcare challenges by combining tissue regeneration with localized and sustained therapeutic effects, paving the way for personalized treatment strategies.^99^^,^^100^ Advances in inkjet, microextrusion, and laser-assisted bioprinting enhance precision, support robust structures, and ensure high cell viability.^101^ Hydrogels such as alginate, gelatin, and polyethylene glycol enable controlled drug release in these applications.^102^ Do et al. employed 3D printing to create a drug release system featuring a poly (lactic-co-glycolic acid) core encased in an alginate shell, demonstrating that the construct is non-toxic to human embryonic kidney cell line or bone marrow stromal stem cells.^102^ Stimuli-responsive bioinks, triggered by pH, temperature, or light, allow targeted drug release,^103^ while nanoparticle integration enhances precision and sustained delivery. These constructs have applications such as localized cancer therapy, delivering chemotherapeutics to tumors with reduced systemic toxicity.^104^^,^^105^ In wound healing, scaffolds embedded with growth factors and antibiotics accelerate tissue repair and combat infections.^106^ Specific biomaterials used as scaffolds in tissue engineering demonstrate intrinsic antimicrobial properties. This capability facilitates the formation of a cellular microenvironment that not only enhances cellular responses but also successfully curtails microbial growth.^107^ Orthopedic applications benefit from constructs that release osteogenic factors, promoting bone and cartilage regeneration. Studies in cells and animals indicate that aspirin may support bone health by promoting the survival and differentiation of osteoblast precursor stem cells. However, its therapeutic use is hindered by issues of acquired resistance and cytotoxicity. Therefore, Li et al. developed polycaprolactone based bioactive composite scaffolds loaded with aspirin liposomes to promote osteogenesis and immunomodulation of human mesenchymal stem cells.^107^ Zhang et al. fabricated 3D composite scaffolds using Fe_3_O_4_ nanoparticles, mesoporous bioactive glass and polycaprolactone 3D-printing technique. Doxorubicin was incorporated in the composite scaffolds, which exhibited a sustained drug release for local drug delivery application in primary bone tumors and bone metastasis.^108^

#### Integration with stem cell therapy

The integration of 3D-printed scaffolds with stem cell^108^ therapy represents a significant advancement in the field of regenerative medicine, offering enhanced approaches for tissue repair and localized drug administration.^109^ These scaffolds act as supportive frameworks that promote the adhesion, proliferation, and differentiation of stem cells, while also incorporating drug delivery systems to ensure targeted therapeutic effects.^110^^,^^111^ These 3D-printed scaffolds have wide applications in tissue repair, including bone,^112^ cartilage,^110^ and skin,^113^ as well as more complex tissues such as cardiac^114^ and neural regeneration.^115^ Cell-laden scaffolds are developed through three approaches: scaffold-based, scaffold-free, and synergistic. The scaffold-based method provides structural support but struggles with cell density and vascularization. The scaffold-free method promotes natural tissue formation but lacks stability. The synergistic approach combines both for enhanced tissue engineering outcomes.^116^

Advancements in stem cell research, especially with induced pluripotent stem cells, are driving progress in tissue-engineered skin substitutes, overcoming ethical concerns linked to embryonic stem cells and enabling skin regeneration.^117^ Embedding neural cells into 3D-printed scaffolds enhances cell differentiation, growth, and simulates *in vivo* conditions for studying neural development and network formation.^118^ The research conducted by Koroleva et al. involved the development of functional neuronal networks from human-induced pluripotent stem cells (hiPSCs) through the use of laser-based 3D scaffolds made from a biocompatible resin called Dental LT Clear. Throughout a duration of 120 days, the iPSC-derived neural stem cells replicated the characteristics of cortical stem cells, differentiating into excitatory and inhibitory neurons, and successfully forming functional synapses and neural circuits.^119^

Cell-laden printing techniques enable the creation of biomimetic human-like tissues by encapsulating stem cells in biomaterials that foster tissue growth.^120^ When these stem cells are combined with 3D-printed scaffolds, they are placed in a microenvironment that mimics natural extracellular matrices, enhancing their regenerative potential.^121^ Research by Gruene et al. demonstrated that mesenchymal stem cells can survive the printing process and retain their ability to differentiate into bone-forming cells.^122^ Additionally, advanced bioinks that include growth factors and nanoparticles improve scaffold functionality by allowing controlled release of therapeutic agents, promoting tissue regeneration while addressing inflammation and infections.^123^ Shafiee et al. developed a medical-grade wound dressing from Poly-ε-caprolactone fibers infused with human gingival mesenchymal stem cells, which significantly improved wound healing and reduced scar formation in a rat model over six weeks.^124^

#### Relevance to pediatric and geriatric populations

The advancements in 3D bioprinting hold particular promise for pediatric and geriatric patients who often present unique challenges in regenerative medicine. For pediatric patients, bioprinting enables the fabrication of patient-specific implants and scaffolds to address congenital defects, pediatric bone tumors, or traumatic injuries, while minimizing the need for invasive donor-site harvesting.^125^ In geriatric populations, bio-printed constructs can help regenerate tissues compromised by age-related degeneration, such as osteoarthritic cartilage, chronic wounds, or bone defects, where traditional therapies may be less effective. By aligning bioprinting strategies with the physiological and regenerative needs of these vulnerable groups, personalized tissue-engineered solutions can be developed to improve healing outcomes and enhance the quality of life.^126^^,^^127^

## Advanced materials in 3D printing

Advanced 3D printing materials enable personalized implants and precise drug delivery for improved treatments.

### Novel and smart polymers

Smart polymers, with stimuli-responsive properties, enable controlled drug delivery and sustained release.^128^ Hydrogels such as thermo-responsive poly(N-isopropylacrylamide), pH-responsive poly (acrylic acid), and ion-responsive polyelectrolytes have versatile applications in drug delivery, tissue engineering, sensors, and self-healing materials^129^ pH-responsive hydrogels adjust their swelling or shrinking behavior based on pH, enabling site-specific drug release. For example, poly(2-dimethylamino) ethyl methacrylate swells at pH below its pKa due to protonation and shrinks above its pKa due to deprotonation.^130^ A study by Bonkovoski et al. developed a polyelectrolyte complex of poly(2-dimethylamino) ethyl methacrylate and chondroitin sulfate, exhibiting hydrophilic-hydrophobic transitions at pH 6.0, 7.0, and 8.0, unlike non-complexed poly(2-dimethylamino) ethyl methacrylate (transition only at pH 8). The polyelectrolyte complex released chondroitin sulfate in alkaline (pH 8) but not acidic (pH 6) conditions, with thermo-responsive behavior influenced by pH-dependent lower critical solution temperature reduction.^130^ Poly(N-isopropylacrylamide)-based hydrogels are thermo-responsive, transitioning at 32°C to enable controlled drug release and applications in tissue regeneration and artificial muscles.^129^ Matsumura et al. developed a thermos responsive, injectable composite gel using N-isopropylacrylamide for thermal sensitivity, 2-hydroxyethyl methacrylates for hydrophilicity, and methacrylate polylactide for biodegradability. The gel synthesized via free radical polymerization, solidifies at body temperature, mimics myocardial mechanics, and supports tissue remodeling for regenerating functional cardiac tissue.^131^

Light-triggered hydrogels enable controlled drug delivery through structural changes induced by UV, visible, or infrared light.^132^ Incorporating photosensitive components such as azobenzene or photocleavable proteins allows controlled release, making them effective for applications such as chemotherapy and wound healing.^133^^,^^134^ Qiu et al. developed a black phosphorus nanocomposite agarose hydrogel with doxorubicin, which enhances drug diffusion and therapeutic efficiency under near-infrared light exposure.^135^

Redox-responsive hydrogels enable targeted drug release by reacting to redox stimuli such as glutathione and hydrogen peroxide.^136^ Zhao et al. developed gelatin/silica-aptamer nanogels for gene delivery, releasing siRNA in nucleolin-positive A549 cells upon glutathione stimulation.^136^ Enzymatically responsive hydrogels enable targeted drug delivery.^137^ Yang et al. developed nanogels cross-linked with cinnamyloxy groups in PEGylated hyaluronic acid, which specifically respond to hyaluronidase. These nanogels remain stable under various biological conditions but degrade rapidly in tumor cells with high hyaluronidase levels, facilitating the swift release of cytochrome *c*.^138^

Multi-responsive hydrogels, reacting to stimuli such as temperature, light, pH, and redox conditions, allow precise drug release.^139^ Gao et al. created thermo-photo-redox-responsive nanogels,^140^ while Jo et al. developed a hydrogel responsive to pH, reducing agents, oxidants, and NIR light for controlled doxorubicin release and reduced side effects.^141^

### Hydrogels

3D-printed hydrogels enable personalized drug delivery with customizable structures, enhanced biocompatibility, and support for multi-drug therapies, ensuring precise, sustained, and targeted therapeutic outcomes.^142^ Hydrogels are classified into three types: natural (biopolymers such as alginate, gelatin, collagen, and offering biocompatibility), synthetic (materials such as PEG, PVA, and polyacrylamide, with tunable properties and strength), and composite (blends of natural and synthetic materials, optimizing functionality and stability for advanced biomedical uses).^143^^,^^144^

**Figure 2:**
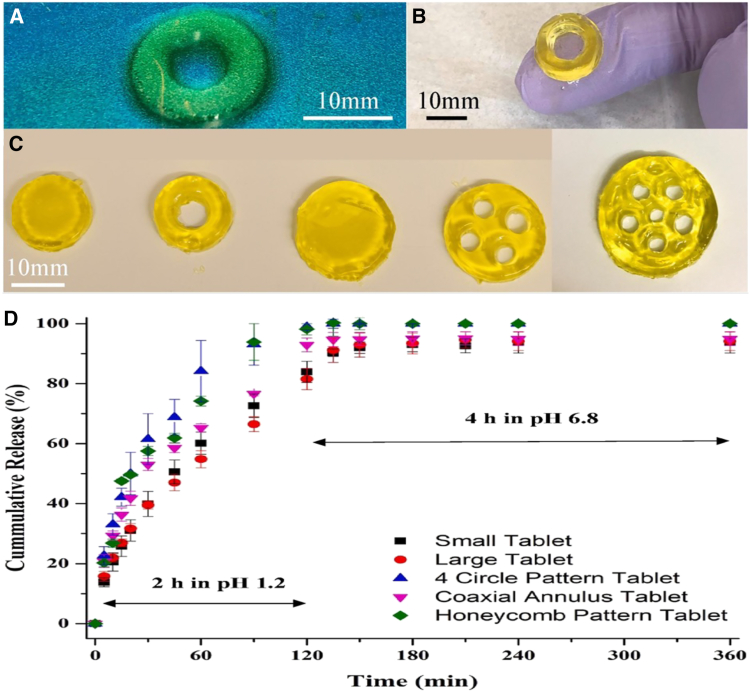
3D-printed coaxial annulus hydrogel tablets (A–D) (A) coaxial annulus hydrogel tablet during 3D printing, showing 3 layers, (B) 3D-printed coaxial annulus hydrogel tablet on a human finger, (C) various 5-mm thick 3D-printed samples: small tablet (15 mm), coaxial annulus (15-mm outer, 10-mm inner), large tablet (20 mm), 4-circle pattern (20 mm with 4-mm holes), and honeycomb pattern (20-mm with hexagonal holes, and (D) *in vitro* cumulative release profiles of ascorbic acid from 3D-printed tablets with varying shapes and sizes. Adapted with permission from Karakurt et al.,^145^ Copyright 2020, Elsevier.

To facilitate the progress of personalized medication development, Karakurt et al.^145^ created customized tablet geometries utilizing SLA 3D printing techniques. Their strategy involved a groundbreaking biocompatible photochemical system that incorporated ascorbic acid encapsulated within a polyethylene glycol dimethacrylate (PEGDMA)-based polymer matrix, which was polymerized with riboflavin serving as a photoinitiator. Through the optimization of process parameters, they successfully created the structures illustrated in Figures 2A–2C, featuring a standard layer thickness of 200 μm and an X-Y resolution of less than 1 mm. *In vitro* experiments were conducted to assess the release of ascorbic acid under conditions mimicking gastric fluid (pH 1.2) for the initial 2 h, followed by simulated intestinal fluid (pH 6.8) for the subsequent 4 h at a temperature of 37 °C. Within the first 15 min, the hydrogels released around 15% of the encapsulated ascorbic acid into the gastric medium. The release rate gradually increased, achieving equilibrium in the intestinal fluid by the end of the 6-h experiment. The geometry and dimensions of the hydrogel tablets influenced the release profile, as illustrated in Figure 2D. Notably, after 60 min of the release period, the honeycomb and coaxial annulus tablet gels demonstrated higher release rates (approximately 80%) compared to other samples (around 60%) with statistical significance (*p* < 0.05). This trend persisted throughout the 6-h duration. The rate of ascorbic acid release ranked as follows: honeycomb/coaxial annulus tablet > 4-circle tablet > small tablet/large tablet. In this study, a larger surface area-to-volume ratio contributed to a more effective release from the carrier.^145^

Osteochondral defects, caused by trauma, degeneration, or congenital issues, challenge orthopedics.^146^ Effective scaffolds require biomimetic materials and growth factors such as bone morphogenetic protein 2 to support osteoblast differentiation and cellular activity.^147^

**Figure 3:**
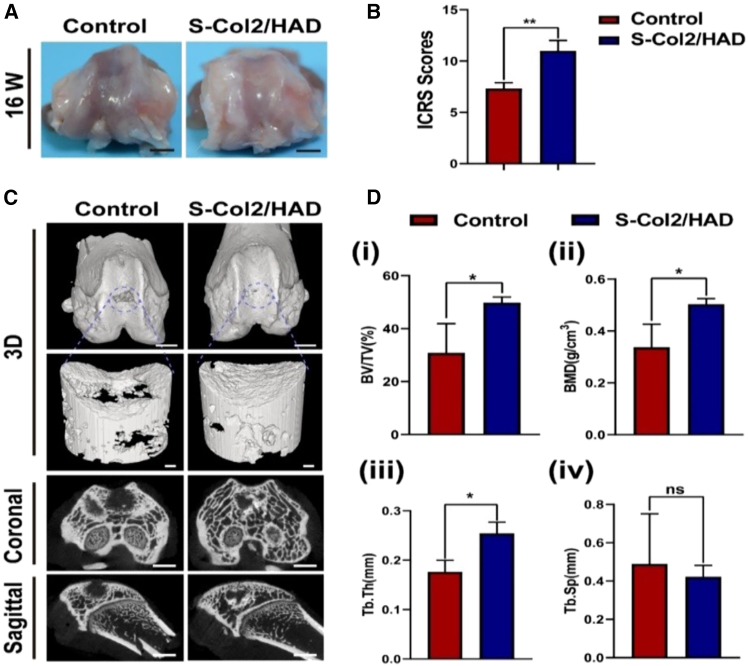
Osteochondral repair with 3D-printed hydrogel scaffolds in rats (A–D) (A) macroscopic images at 16 weeks; control group lacked scaffolds, scale bar: 2 mm, (B) macroscopic scoring (*n* = 5), (C) micro-CT showing new bone morphology, scale bars: 2 mm and 20 μm (ROI), and (D) quantification of BV/TV (i), BMD (ii), Tb.Th (iii), and Tb.Sp (iv) from micro-CT, analyzed by ANOVA. Adapted with permission from Wu et al.,^148^ Copyright 2024, Elsevier.

Wu et al. developed hydrogel scaffolds for sustained release of bone morphogenetic protein 2 (BMP-2) and collagen type II to support cell differentiation into chondrocytes and osteoblasts. The 3D-printed scaffold (S-Col2/HAD) featured a dual-layer design: the upper layer included sodium alginate, methacrylated hyaluronic acid, collagen type II, and calcium, while the lower layer incorporated dopamine-modified hyaluronic acid, BMP-2, and calcium. Implanted in the femoral trochlea of rats, the S-Col2/HAD scaffold demonstrated superior cartilage repair, with tissue resembling natural cartilage, compared to cracks and poor integration in controls (Figure 3A). ICRS scoring also favored the treatment group (Figure 3B). Micro-CT scans showed near-complete bone filling (Figure 3C), increased bone volume fraction (BV/TV), higher bone mineral density (BMD) (Figure 3 di,ii), and improved trabecular morphology in the treated group (Figure 3 diii,iv), confirming the scaffold’s role in enhancing osteogenesis and chondrogenesis.^148^

### Bioinks

#### Functional bioinks

**Figure 4:**
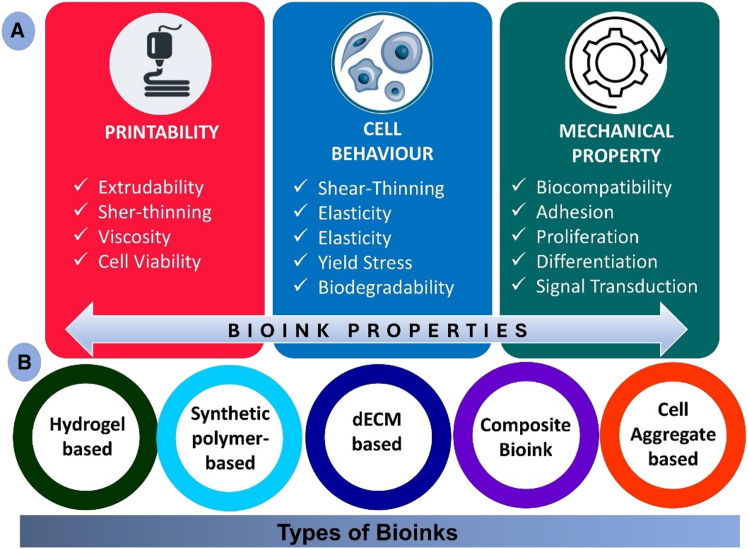
Bioink properties and types for 3D priniting (A) Bioink properties include printability, cell behavior, and mechanical performance. (B) Bioink types comprise hydrogel-based systems, synthetic polymer-based systems, decellulized extracellular matrix (dECM)-based materials, composite bioinks, and cell aggregate-based constructs.

Bioinks are cell-laden materials for 3D bioprinting, used to create tissue-like structures for tissue engineering, regenerative medicine, and drug testing.^149^ Derived from natural, synthetic, or hybrid materials, they are categorized into hydrogel-based (e.g., protein/polysaccharides), dECM-based (native tissue mimicry), synthetic polymer-based (custom mechanical properties), cell aggregate-based (complex tissue formation), and composite bioinks (optimized mechanical and biological properties).^150^ The Figure 4 illustrates the different types of bioinks and their properties.

Decellularized extracellular matrix (dECM) bioinks, rich in collagen, mimic native tissue environments, enhancing cell survival, differentiation, and therapeutic outcomes. Research indicates that hydrogels based on decellularized extracellular matrix derived from cardiac tissue (hdECM) are effective in alleviating cardiac hypertrophy and fibrosis by influencing inflammation, apoptosis, and cardiac metabolism.^151^

**Figure 5:**
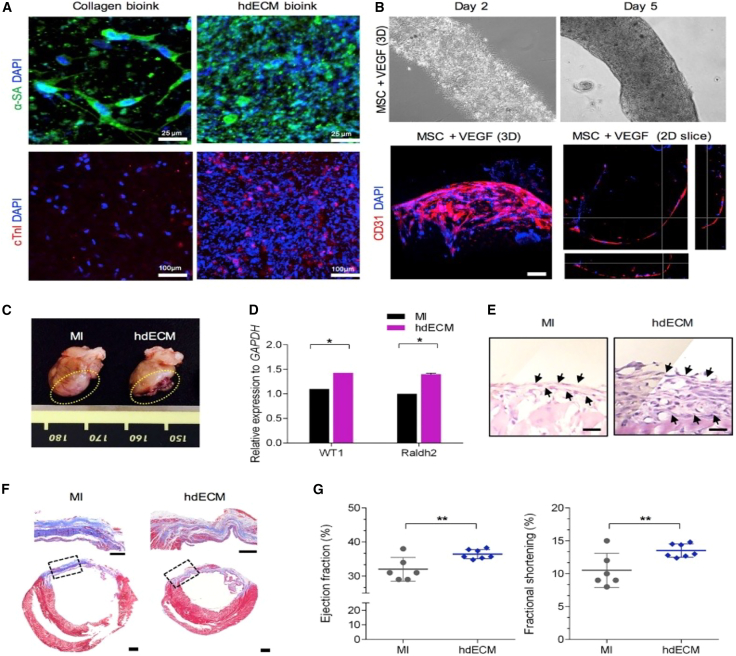
Functional analysis of hdECM bioink (A–G) (A) immunofluorescence for α-sarcomeric actin (green) and cardiac troponin I (red), (B) microscopic imaging, (C) optical images of sacrificed samples, (D) epicardial activation-related gene expression, (E) histological sections of the epicardium, (F) Masson’s trichrome staining, and (G) quantitative analysis. Adapted with permission from Jang et al.,^151^ Copyright 2017, Elsevier.

Jang et al. developed a 3D pre-vascularized stem cell patch using hdECM-based hydrogel bioink via 3D printing, combining cardiac progenitor and mesenchymal stem cells.^151^ The hdECM bioink (Bioink I) significantly enhanced cardiac progenitor maturation compared to single-component bioinks such as collagen hydrogel (Figure 5A). Bioink II, containing mesenchymal stem cells and vascular endothelial growth factor, promoted vascular structure formation, including hollow tube development after five days (Figure 5B). Seven days post-implantation, the hdECM group showed reduced eccentric heart remodeling compared to the MI control group (Figure 5C). Enhanced epicardial activation was evidenced by the elevated expression of Wilms tumor protein 1 (WT1) and retinaldehyde dehydrogenase 2 (Raldh2) genes (Figure 5D) and substantial epicardial expansion, indicating epithelial-mesenchymal transition (Figure 5E). After eight weeks, the hdECM group exhibited decreased cardiac remodeling and fibrosis (Figure 5F) along with improved left ventricular remodeling, ejection fraction, and fractional shortening, whereas the MI group showed progressive functional decline (Figure 5G).

**Figure 6:**
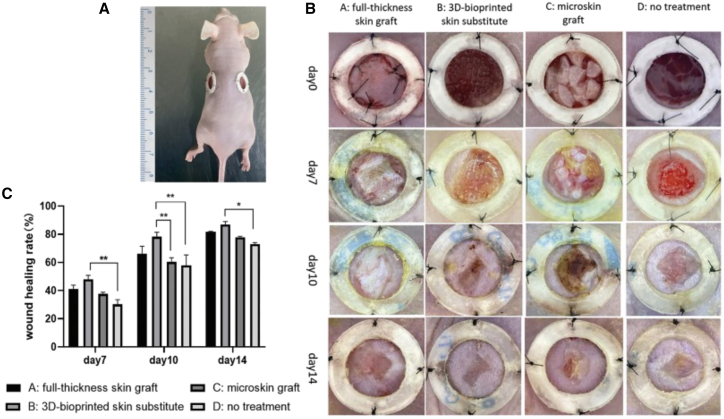
Evaluation of *in vivo* wound healing progression and treatment efficacy in a rat model (A–C) (A) *in vivo* wound healing rat model, (B) representative images of wound progression in each group on days 0, 7, 10, and 14 post-operations, and (C) wound healing rate (%) across groups at different time points. Adapted with permission CC BY4.0 from Fu et al.,^153^ Copyright 2023, ACCSCEINCE, Creative Commons Attribution 4.0.

Adipose tissue-derived dECM, rich in peptides and glycosaminoglycans, supports cell accumulation, proliferation, and differentiation, promoting angiogenesis and tissue repair. Its self-assembling hydrogel properties make it a promising treatment for wound healing.^152^ To improve the printability of dECM pre-gel, Fu et al. created a composite hydrogel bioink by integrating methacrylated gelatin (GelMA), methacrylated hyaluronic acid (HAMA), and dECM pre-gel. This innovative composite hydrogel was used to produce 3D-printed tissue-engineered skin substitutes infused with human adipose-derived stem cells (hADSCs), aimed at assessing its effectiveness in facilitating wound healing.^153^ In a rat model, 3D-printed skin substitutes with human adipose-derived stem cells (hADSCs) showed faster wound healing compared to full-thickness grafts, microskin grafts, and controls (Figure 6A). By day 14, the 3D-bioprinted group achieved full closure with minimal scarring, outperforming other treatments (Figures 6B and 6C).^153^

**Figure 7:**
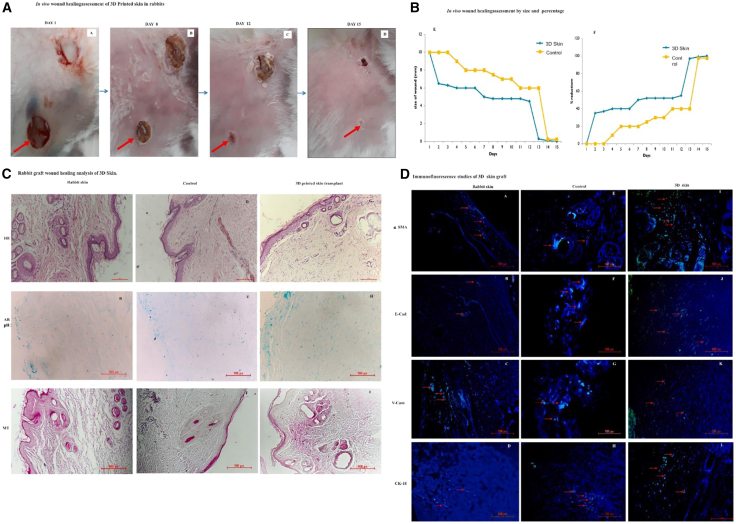
*In vivo* wound healing activity (A–D) (A) assessment of artificial skin in rabbits, (B) assessment by size and percentage, (C) histological analysis, and (D) immunofluorescence study of 3D skin graft. Adapted with permission from Damle et al.,^154^ Copyright 2024, Elsevier.

Tissue engineering and 3D printing combine biomaterials and living cells to develop advanced biological dressings that mimic the extracellular matrix, enhancing wound healing and regeneration. Using hydrogels, collagen, and patient-specific cells, these technologies provide customizable and biocompatible solutions for complex wounds. Damle et al. designed a skin-specific bioink by integrating digested chicken skin with polyvinyl alcohol (PVA) and gelatin, enabling the creation of 3D-printed skin patches.^154^ The healing of rabbit wounds is illustrated in Figure 7A for days 0, 8, 12, and 15. In the first week, the wounds treated with 3D-printed skin showed a reduction of 30–40%, whereas the control group experienced a 20% reduction. By day 15, the wounds with 3D-printed skin had fully healed, in contrast to the control wounds, which only achieved a 90% reduction in size (Figure 7B). Histological analysis (Figure 7C) revealed enhanced epithelialization and neovascularization in transplanted skin samples compared to controls. Immunohistochemistry (Figure 7D) identified several biomarkers, including α-smooth muscle actin (α-SMA), E-cadherin (E-CAD), Vascular cell adhesion molecule (V-CAM-1), and Cytokeratin 18 (CK-18). These findings corroborate the enhanced processes of epithelialization, neovascularization, and the recruitment of smooth muscle cells in wounds that received treatment with 3D-printed skin.

**Figure 8:**
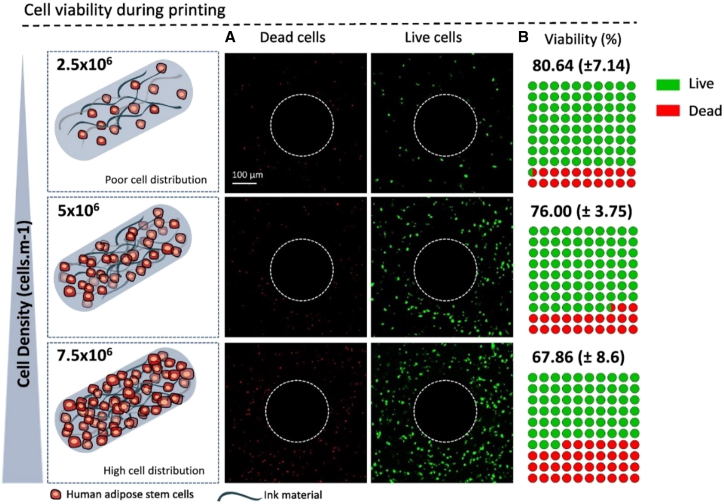
hASC viability during 3D printing (A and B) (A) confocal pictures show live (green) and dead (red) cells at varying densities, and (B) quantitative analysis performed with ImageJ. Adapted with permission from Diogo et al.,^155^ Copyright 2022, Elsevier.

The 3D printing system was tested using human adipose-derived stem cells (hASCs), which possess multipotent capacities to stimulate new tissue growth. Recently, Diogo and his colleagues have put forward a bioactive bioink that combines mineralized shark collagen, alginate, and human adipose stem cells for the purpose of bone tissue regeneration. They created a hydrogel bioink by employing mineralized shark collagen along with sodium alginate to effectively encapsulate the stem cells.^155^ When cells are packed tightly together, they interact more frequently, which can overwhelm them and deprive them of oxygen. Therefore, an adequate cell distribution within the printed filaments is required to enhance cell-to-cell interactions.^156^ Cell viability of the fabricated hydrogel bioink was assessed at three different cell densities (Figure 8A). The lowest cell concentration (2.5×10^6^ cells/mL) led to uneven cell distribution but improved cell viability during the printing process. The intermediate density provided good cell distribution and survival. However, a higher cell density (7.5×10^6^ cells/mL) led to lower cell survival, as increased cell contacts during nozzle passage caused higher cell death, as shown in Figure 8B. These results align with expectations, as higher cell densities increase cell-wall contact, leading to greater cell loss.^155^

#### Customization and scalability

The development of scalable and customizable bioinks is crucial for advancing 3D printing in regenerative medicine.^156^ Significant challenges include ensuring the materials’ biocompatibility, optimizing their viscosity for efficient printing, and achieving a balance between the mechanical properties and biological functions. Moreover, the need to scale production while preserving quality and accommodating specific applications complicates the process.^157^ Recent advancements, such as the development of hybrid biomaterials, modular bioink systems, and high-throughput manufacturing techniques, are addressing these issues.^158^ Additionally, improvements in bioprinting technology, AI-driven design, and the incorporation of omics data are enhancing the potential for customization.^159^^,^^160^ Scalable cell culture systems and sustainable practices further support large-scale applications. Together, these innovations position bioinks as transformative tools for PM, tissue engineering, and drug development, promising significant progress in healthcare.^161^

#### Pediatric and geriatric applications of bioinks

The development of functional bioinks can be strategically directed to address specific age-related clinical needs. In children, soft, adaptable bioinks can be used to produce growing tissue scaffolds that accommodate the dynamic anatomical changes of the developing body, providing regenerative options for congenital anomalies or pediatric reconstructive surgery. For elderly patients, bioinks designed with osteo-inductive, angiogenic, or antimicrobial properties can enhance bone regeneration, accelerate wound closure, and reduce infection risk, all of which are crucial for managing age-related tissue deterioration and comorbidities. By tailoring bioinks for these demographic groups, 3D bioprinting can play a transformative role in delivering safe, patient-centered regenerative therapies.^162^^,^^163^^,^^164^

## Challenges and future directions

While 3D printing technology has distinct benefits and has made significant advancements, its use in the pharmaceutical sector^165^ is still in its infancy. It continues to encounter challenges such as quality control issues, a lack of available polymer excipients, technological limitations, and insufficient regulatory frameworks.

### Regulatory hurdles

The incorporation of 3D printing technology into pharmaceutical development has the potential to transform the industry, offering bespoke and tailored drug delivery solutions. However, this innovation also presents significant regulatory hurdles.^166^ Key milestones include the FDA’s clearance of the first 3D printed cranial implant in 2013 and a titanium spinal implant. However, no 3D-bioprinted tissues or organs have received FDA approval due to a lack of standardization in technology, materials, and processes, which hinders their clinical adoption. Additionally, the complexity of 3D-bioprinted cell-laden tissues presents challenges for regulatory authorities compared to traditional 3D printed devices.^167^ The intricate nature of 3D printing, combined with the diverse range of printing methods and materials employed, raises concerns about the safety, efficacy, and quality control of 3D-printed pharmaceutical products.^168^ Regulatory bodies have initiated efforts to address these challenges, but the lack of clear and comprehensive guidelines for 3D-printed medicines complicates the approval and oversight processes.^169^ Although there have been improvements in the regulation of 3D printed medical devices, the FDA has yet to approve any 3D-bioprinted tissues or organs. The absence of standardization in technology, materials, and processes restricts their use in clinical settings. Furthermore, the intricate nature of 3D-bioprinted cell-laden tissues poses additional challenges for regulators compared to conventional devices. While practices such as tracking cell sources, evaluating cell viability, and maintaining sterility can be adopted, logistical issues and uncertainties regarding long-term safety in humans continue to be major obstacles.^170^

3D-printed medicines must meet the same regulatory standards as other drugs, but only one solid oral dosage form has been approved for market use due to ongoing challenges, particularly the lack of established regulatory pathways.^171^ While the FDA provided guidance for 3D-printed medical devices in 2017, it does not fully address pharmaceuticals, leaving the regulatory framework for 3D-printed drugs underdeveloped and lacking specific guidelines.^172^ The lack of standardized protocols for assessing 3D-printed drugs creates challenges in ensuring consistency, reproducibility, and quality. To address these regulatory issues, it is vital to create specific guidelines for 3D-printed pharmaceuticals that consider the unique characteristics of the technology. Manufacturers should implement strong quality control measures, including process validation and real-time production monitoring. Collaboration among industry experts, regulatory bodies, and academic researchers is essential to develop evidence-based frameworks that tackle the distinct challenges of 3D printing, ensuring that these products meet the required standards for market entry.^172^

Currently, only a limited number of printers are capable of functioning in GMP^173^ conditions, and their high prices restrict their use in clinical environments. Furthermore, it is essential for healthcare professionals to undergo appropriate training to produce personalized medications that meet stringent quality standards. To facilitate the effective transition of knowledge from research to clinical practice, it is essential to optimize protocols that reduce variability between production batches.^174^ Currently, only a limited number of clinical trials are investigating the use of 3D-printed tablets^175^ to treat specific conditions for which no commercially available medication exists. The primary reasons for this issue include the complexity of operating 3D printers at high-quality standards, the absence of well-defined regulatory guidelines, a shortage of skilled personnel, and limited access to GMP-compliant printers^176^ that avoid cross-contamination.^177^

### Technical limitations

3D printing in pharmaceuticals faces several challenges, particularly in resolution, precision, scalability, and bioink development.^178^ High resolution and precision are essential for producing dosage forms with accurate dimensions and consistent drug release profiles.^179^ However, limitations in printer capabilities, including nozzle diameter, material flow consistency, and movement precision, can lead to variability in dosage and therapeutic efficacy. Advanced techniques such as stereolithography offer improved resolution compared to traditional methods such as fused deposition modeling, but their high cost, slow speed, and limited material compatibility restrict their practical application.^179^ Additionally, achieving uniform drug distribution in multi-drug systems remains difficult due to incomplete material mixing and extrusion inconsistencies. The final formulation may exhibit configurational and surface irregularities that need to be addressed by optimizing various manufacturing parameters^180^ Scaling up 3D printing from laboratory to industrial production adds another layer of complexity. Current 3D printing processes, including fused deposition modeling and stereolithography, rely on slow, layer-by-layer approaches that are not conducive to mass production. This makes it challenging to meet the throughput required for large-scale manufacturing.^181^ Minor variations in environmental conditions, equipment settings, or material properties during industrial production can lead to inconsistencies in product quality. Furthermore, integrating 3D printing into pharmaceutical manufacturing requires compliance with Good Manufacturing Practices, which demands extensive validation. High costs associated with industrial-scale 3D printers, pharmaceutical-grade materials, and post-processing steps, such as curing and sterilization, further hinder scalability. Innovations such as parallelized printing systems and high-speed continuous manufacturing are needed to address these issues.^182^

Bioinks, a key component in bioprinting, present additional limitations. Many bioinks lack the mechanical strength to maintain complex shapes after printing, often resulting in deformation or collapse. The limited availability of medical-grade materials for 3D printing poses a challenge in the development of safe and effective products. Achieving uniform drug distribution within bioinks is also challenging, with active ingredients potentially segregating or degrading during printing. Furthermore, bioinks must comply with strict sterilization, storage, and regulatory requirements, making their large-scale application difficult.^183^ In conclusion, addressing these limitations is critical for unlocking the full potential of 3D printing in pharmaceuticals, enabling its use for innovative drug delivery systems and PM.

The potential of 3D printing for various medical applications has been extensively demonstrated. However, much of the existing research has been conducted using standard 3D printers that do not meet regulatory standards. Standard 3D printers might lack the resolution to create intricate dosage forms with precise drug distribution.^184^ Specialized high-resolution printers would likely be needed for PM applications. The materials used in 3D printing can impact the achievable resolution and precision.^185^ There is no guarantee that cross-contamination will not occur during the production of various dosage forms. This issue is particularly evident with FDM printers,^186^ where different material combinations are processed through the same extrusion nozzle, making thorough cleaning difficult. Some materials might be more difficult to extrude or solidify with fine detail, requiring adjustments to the printing parameters and potentially limiting the complexity of achievable designs. Maintaining consistent resolution and precision across large-scale production requires stringent process control. Factors such as temperature fluctuations, material flow rates, and printing speed can all affect the final product’s quality and accuracy.

Scaling up the production of 3D printed PM from a laboratory setting to an industrial scale also presents significant challenges. One of the main issues is the need for robust and reliable manufacturing processes that can consistently produce high-quality medical products on a large scale.^187^ This requires investments in advanced 3D printing equipment, automation technologies, and quality control systems. Additionally, ensuring the biocompatibility and safety of 3D-printed medical devices, especially when using novel materials or intricate designs, is crucial for regulatory approval and patient well-being. Denis et al.^188^ developed a novel 3D printing platform within a hospital setting. This platform is designed to produce personalized medications tailored for patients participating in the OPERA clinical trial. Semi-solid extrusion 3D printing was initially used to create a tamoxifen pharmaceutical ink, formulated in alignment with French compounding regulations. Subsequently, an innovative pellet-dispensing printhead facilitated the incorporation of commercial pellets containing venlafaxine or duloxetine. These medications were successfully produced and developed by the clinical pharmacy department at the Gustave Roussy Cancer Hospital in Paris, France. This study illustrates the potential for formulating and manufacturing tamoxifen combination medications in a hospital environment using a pharmaceutical 3D printer. This approach aims to support clinical trials that demand a high production rate for these medications.

### Future research areas

The future of 3D printing in PM is set to be shaped by a convergence of advanced manufacturing, data-driven design, and biological innovation. One of the most promising developments is the integration of AI and machine learning into the design, optimization, and quality control of 3D printed drug delivery systems and tissue-engineered constructs.^189^ AI algorithms can analyze vast datasets from genomics, patient health records, pharmacokinetics, and material science to generate highly individualized drug formulations or implant designs. For example, ML models can predict optimal release kinetics based on patient-specific parameters and guide the selection of suitable materials and 3D printing parameters to achieve desired therapeutic outcomes.^190^

In drug delivery, AI-enabled simulations can streamline the development of complex dosage forms, such as polypills with variable release profiles or microneedle arrays for transdermal delivery.^191^ In regenerative medicine, AI can assist in optimizing scaffold geometry, mechanical properties, and cell distribution to enhance tissue regeneration and integration. Moreover, AI-based image analysis and digital twin technologies could enable real-time monitoring and adaptive control of the bioprinting process, improving precision and reproducibility in clinical applications.^192^

Coupled with decentralized manufacturing models, AI could also facilitate on-demand drug printing at local pharmacies or hospitals by recommending dosage adjustments based on real-time patient data. This level of responsiveness would mark a shift from mass production to individualized therapeutics, making personalized healthcare more accessible and scalable.^193^ However, the successful integration of AI into pharmaceutical 3D printing and bioprinting requires a robust digital infrastructure, interdisciplinary collaboration, and the development of regulatory frameworks that ensure transparency, safety, and reproducibility.^194^ Future research should focus on validating AI-generated designs through preclinical and clinical studies and creating standardized datasets to train predictive models. As these technologies mature, the fusion of AI with 3D printing will enable a new paradigm in precision medicine where therapeutic solutions are not only patient-specific but also dynamically optimized in real-time.^195^

### Critical analysis and comparative perspectives

While significant advances have been made in applying 3D printing technologies to PM and complex drug delivery, each technique has distinct strengths and limitations that shape its practical applicability. For example, Fused Deposition Modeling (FDM) is cost-effective and widely accessible for producing solid oral dosage forms, but it is limited to thermally stable drugs and simpler geometries.^25^^,^^65^^,^^66^ In contrast, SLA and DLP provide superior resolution and the capability to fabricate intricate microneedles, implants, and scaffolds.^65^^,^^66^^,^^74^ However, they are constrained by the availability and safety of photopolymer resins and potential photochemical interactions with active pharmaceutical ingredients.^44^^,^^45^^,^^46^

Inkjet-based 3D printing enables precise dosing and is useful for localized drug deposition and coating.^26^^,^^78^ Nevertheless, it typically accommodates only low-viscosity formulations and may not be suitable for high drug loads or complex, multi-drug structures. Bioprinting, which combines cells and bioinks to create tissue-like constructs, holds transformative potential for regenerative medicine and localized drug delivery.^149^ However, issues such as ensuring cell viability during printing, achieving sufficient mechanical stability, and navigating complex regulatory pathways remain significant obstacles to clinical translation.^167^

Critically comparing these approaches shows that no single method is universally ideal; instead, the choice of technique must align with the intended therapeutic application, drug properties, patient-specific requirements, and scalability needs. Despite promising prototypes and preclinical successes, large-scale translation is still challenged by difficulties in maintaining consistent quality, meeting GMP standards, and managing the costs and technical demands of advanced printing equipment and medical-grade materials.^169^^,^^173^

To address these limitations, future work should prioritize the development of hybrid or multi-modal printing systems that leverage the complementary strengths of different methods such as integrating FDM with inkjet or SLA for producing complex polypills with multiple APIs and controlled-release profiles.^71^ Moreover, robust, standardized evaluation protocols are needed to assess long-term safety, efficacy, and patient adherence, particularly for personalized dosage forms and implantable systems.

Overall, this critical perspective underscores that realizing the full promise of 3D printing in PM will require not only technological and material innovation but also interdisciplinary collaboration and progressive regulatory frameworks that facilitate safe, reproducible, and scalable production of customized therapeutic solutions.

## Conclusion

In conclusion, 3D printing technology is revolutionizing the pharmaceutical industry by enabling the creation of customized dosage forms tailored to individual patient needs. With its capacity for producing patient-specific medications and polypills, 3D printing offers significant improvements in PM, especially in pediatrics and geriatrics, where dosage forms can be adjusted for size, shape, and flavor to enhance compliance. Case studies and clinical trials have shown promising outcomes in the efficacy and patient adherence of 3D-printed medicines. The technology also allows for the development of complex drug delivery systems, including multi-layered tablets for controlled release and microscale and nanoscale drug delivery structures that target specific tissues. The application of 3D printing in drug-eluting implants and biocompatible scaffolds further demonstrates its potential in regenerative medicine, particularly when integrated with stem cell therapy. Advancements in materials such as smart polymers, hydrogels, and bio-inks are driving innovation in drug delivery and bio-printing, but challenges remain. Regulatory hurdles, technical limitations in resolution and scalability, and the need for interdisciplinary collaboration continue to shape the future of 3D printing in pharmaceuticals. However, ongoing research and technological advancements are paving the way for new possibilities in PM and advanced therapeutic solutions. The future of 3D printing in PM and complex drug delivery systems holds immense promise. Advancements in materials, bio-printing, and precision manufacturing will enable more customized therapies, targeted drug delivery, and patient-specific implants. Ongoing research and interdisciplinary collaboration will drive innovation, improving treatment outcomes and patient care.

## Acknowledgments

The authors acknowledge the financial support provided by the 10.13039/501100017170Thailand Science Research and Innovation under the National Science, Research and Innovation Fund (Thailand), Fiscal Year 2568. We also extend our sincere appreciation to the 10.13039/501100024252Faculty of Pharmacy, 10.13039/501100006436Silpakorn University, Thailand, for its invaluable support, which greatly contributed to the successful completion of this work.

## Author contributions

Devesh U. Kapoor: writing – original draft. Anil Pareek: writing – original draft. Priyanka Uniyal: writing – original draft. Bhupendra G. Prajapati: conceptualization and writing – review and editing. Kasitpong Thanawuth: visualization and software. Pornsak Sriamornsak: conceptualization, writing – review and editing, funding acquisition, and project administration.

## Declaration of interests

The authors declare no conflicts of interest.

## Declaration of generative AI and AI-assisted technologies in the writing process

During the preparation of this work, the authors used ChatGPT (OpenAI, 2025) in order to assist with formatting and refining technical language. After using this tool, the authors reviewed and edited the content as needed and take full responsibility for the content of the publication.
